# Co-detection of bocavirus and bacteria in a respiratory specimen from a pregnant woman using multiplex real time PCR; a pathogenic role, or a bystander?

**Published:** 2020-02

**Authors:** Khosrow Agin, Iman Rezaee, Zahra Heydarifard, Seyed-Mohammad Jazayeri

**Affiliations:** 1 Department of Pulmonary and Heart Ward, Loqman Hakim Hospital, Shahid Beheshti University of Medical Sciences, Tehran, Iran; 2 Clinical Virology Research Center, Tehran University of Medical Sciences, Tehran, Iran

**Keywords:** Multiplex real-time polymerase chain reaction, Respiratory co-detection infections, Human bocavirus

## Abstract

A pregnant woman presented by cough and dyspenia. Employing a respiratory multiplex real-time PCR, Human bocavirus (HBoV), Haemophilus influenza and *Staphylococcus aureus* were positive at cycle thresholds (CTs) of 21, 35 and 33.5, respectively. The patient was diagnosed for bacterial respiratory infection superimposed by bocavirus due to a relative high CT value. Patient’s condition improved using bronchodilators and corticosteroid without any further antibiotic treatment. HBoV is not exclusively a bystander pathogen in some patients.

## INTRODUCTION

Community Acquired Pneumonia (CAP) is a common infectious disease with a mortality rate of 2%–14% (1). Apart from bacteria, several respiratory viruses may also cause severe respiratory disease. Despite viral respiratory illnesses are usually self-limiting, however, the emergence of highly virulent strains can lead to high morbidity and mortality. Single or multiplexed molecular assays are recognized as the gold standard for viral respiratory diagnosis (2). The latter which identifies different pathogens simultaneously in one reaction, offers minimal hands-on time and sample preparation along with rapid turnaround time.

The seroprevalence of HBoV is ranged between 76.6% in children and 96% in adults (3, 4). However, the prevalence of HBoV in respiratory specimen ranged from 1% to 56.8% in different age groups at different countries (5). As HBoV has been first identified in clinical samples of the respiratory tract, it has been suggested as an infective agent of respiratory tract. The remaining isolates are more frequently associated with gastrointestinal infections and symptoms. From the beginning of the discovery of this virus, unlike other viruses, HBoV has been recognized as a co-pathogen with more additional pathogens than any other respiratory viruses (6).

Here, we present a case of respiratory illness in a pregnant woman coinfected with triple pathogens detected by a multiplex real-time PCR respiratory panel.

## CASE PRESENTATION

The patient was a 36-years old pregnant woman. She suffered from chronic cough and subsequent dyspnea for five weeks before her admission. The symptoms started with a mild cough which progressively increased in severity and subsequently was associated with dyspnea for the last 2 weeks before the admission. As being diagnosed for acute CAP, she had received empiric antibiotic therapy without any microbiological laboratory assessment and she had completed a course of cefixime (400 mg daily) and azithromycin (500 mg daily) combination five days before the admission.

At the admission time, she was in 34^th^ weeks of pregnancy period. Upon the examination, the patient was ill and anxious. Respiratory rate was 20–22 per minute. General inspiratory and expiratory wheezing was auscultated at both lungs filed. The functional class was III. She was not willing to go to the hospital despite the advice. The patient has anxious for the severity of her disease and the subsequent outcomes of her delivery. Her respiratory rate was decreased to 18–20 per minute using a nebulizer. Taking a chest X-ray was not possible due to her pregnancy. The patient was suspected for the case of bronchial hyper-responsiveness. The patient was treated with inhaler spray corticoid and bronchodilator alone without any antibiotic prescription. A nasopharyngeal aspirate was obtained on the day of her visit and subsequently sent to the laboratory for testing. The nucleic acid was extracted using Viral/bacterial High Pure Nucleic Acid Isolation kit (Roche, Germany) and subsequently was tested by a multiplex real-time PCR using a panel of 33 pathogens including 22 viruses and 12 bacteria (FTD/SIEMENS, Luxembourg). Afterwards, HBoV, Haemophilus Influenza and *Staphylococcus aureus* were positive at cycle thresholds (CTs) of 21, 35 and 33.5. Two days after her admission to the clinic her clinical condition improved.

## DISCUSSION

The presented case was suffered from dyspnea and cough which gradually increased in severity in a few days. As a rule in the country, empirical antibiotic therapy associated with CAP initiates for outpatients even in the absence of diagnostic evaluation. After performing a respiratory multiplex real time PCR, she was positive for two bacteria (*Haemophilus influenza* and *Staphylococcus aureus*) along with bocavirus. The CT values for both bacteria were comparable (33 and 35, respectively). Semi-quantification of the molecular material present in the sample is possible to achieve by molecular techniques (especially real-time PCR), giving additional information about the respiratory microbial load. We believe that the discovery of bocavirus in this patient was not a bystander finding. On the other hand, wheezing and cough were on the favor of bocavirus predominance in the presented patient. In addition, the severity of symptoms could be correlated with the bocavirus superimposed infection in this patient.

The rate of simultaneous co-detection of respiratory pathogens that occur with HBoV (or vice versa) is frequently observed between 60% and 90%. Published data on the prevalence of bocavirus among the Iranian patients with the respiratory symptoms is rare. A prevalence of 6.8% to 10.7% has been reported in children (7–9). Only one published data indicated that bocavirus was detected in 6.6% of adults respiratory specimens (10). Currently, there is no specific approved treatment for HBoV infection. The presented case was treated for clinical wheezing and with probably bronchial hyper responsiveness with inhaler spray corticosteroid and bronchodilator. No, further treatment advised due to her past empiric treatment and also for the detected bocavirus.

In conclusion, HBoV could be a leading cause or an adding cause for the severity of respiratory illness in patients who contain bocavirus coinfection. Application of multiplex real-time PCR will signify our understanding of respiratory infections especially the role of viruses in the initiating or intensification of disease severity.
